# MIAT promotes myofibroblastic activities and transformation in oral submucous fibrosis through sponging the miR-342-3p/SOX6 axis

**DOI:** 10.18632/aging.206121

**Published:** 2024-10-07

**Authors:** Ming-Yi Lu, Chih-Yuan Fang, Pei-Ling Hsieh, Shih-Chi Chao, Yi-Wen Liao, Yoichi Ohiro, Chen-Chia Yu, Dennis Chun-Yu Ho

**Affiliations:** 1 School of Dentistry, Chung Shan Medical University, Taichung, Taiwan; 2 Department of Dentistry, Chung Shan Medical University Hospital, Taichung, Taiwan; 3 School of Dentistry, College of Oral Medicine, Taipei Medical University, Taipei, Taiwan; 4 Division of Oral and Maxillofacial Surgery, Department of Dentistry, Wan Fang Hospital, Taipei, Taiwan; 5 Department of Anatomy, School of Medicine, China Medical University, Taichung, Taiwan; 6 Institute of Oral Sciences, Chung Shan Medical University, Taichung, Taiwan; 7 Department of Medical Research, Chung Shan Medical University Hospital, Taichung, Taiwan; 8 Oral and Maxillofacial Surgery, Division of Oral Pathobiological Science, Faculty of Dental Medicine and Graduate School of Dental Medicine, Hokkaido University, Sapporo, Japan; 9 School of Oral Hygiene, College of Oral Medicine, Taipei Medical University, Taipei, Taiwan

**Keywords:** oral submucous fibrosis, long non-coding RNA, MIAT, miR-342-3p, SOX6

## Abstract

Oral submucous fibrosis (OSF) is an oral potentially malignant disorder that is closely related to the habit of areca nut chewing. Long non-coding RNA (lncRNA) myocardial infarction-associated transcript (*MIAT*) has been identified as an essential regulator in the fibrosis progression. However, the role of *MIAT* in the development of OSF remains unknown. The transcriptomic profile showed that *MIAT* is significantly overexpressed in the OSF cohort, with a positive correlation to fibrotic markers. The silencing of *MIAT* expression in primary buccal mucosal fibroblasts (BMFs) markedly inhibited arecoline-induced myofibroblast transformation. Mechanistically, *MIAT* functioned as a *miR-342-3p* sponge and suppressed the inhibitory effect of *miR-342-3p* on *SOX6* mRNA, thereby reinstating *SOX6* expression. Subsequent RNA expression rescue experiments confirmed that *MIAT* enhanced resistance to apoptosis and facilitated myofibroblastic properties such as cell mobility and collagen gel contraction by regulating the *miR-342-3p/SOX6* axis. Taken together, these results suggest that the abnormal upregulation of *MIAT* is important in contributing persistent activation of myofibroblasts in fibrotic tissue, which may result from prolonged exposure to the constituents of areca nut. Furthermore, our findings demonstrated that therapeutic avenues that target the *MIAT/miR-342-3p/SOX6* axis may be a promising approach for OSF treatments.

## INTRODUCTION

Oral submucous fibrosis (OSF) is predominantly prevalent in the areas of South and Southeast Asia, with a malignant transformation rate into oral squamous cell carcinoma (OSCC) ranging up to 4-13% [1–3]. Recognized as an areca nut-associated oral potentially malignant disorder (OPMD), OSF is characterized by a gradual reduction in mouth opening, largely due to the dysregulation the extracellular matrix (ECM) synthesis and degradation [4]. Despite various approaches being applied to alleviate symptoms [5–8], none have proven curative for OSF. Hence, deeper understanding in the pathogenesis of OSF is necessary to developing more effective therapies and preventing its malignant transformation.

Myofibroblasts, key ECM-secreting cells, play a major role in wound healing and pathological fibrosis. These cells can escape from apoptosis, leading to continued tissue remodeling and fibrosis through increased production of mediators like pro-inflammatory cytokines and transforming growth factor-β (TGF-β), as well as excessive secretion of ECM components such as collagens [9–11]. In OSF, higher expression of myofibroblast markers has been reported [12]. Therefore, approaches to suppress the persistent activation of myofibroblasts offers a promising strategy to resolve pathological fibrosis.

Emerging studies have revealed the role of epigenetic regulation by non-coding RNAs (ncRNAs) in modulating the activation of myofibroblasts [13]. The interplay between long non-coding RNAs (lncRNAs) and microRNAs (miRNAs) has gained great attention for its impact on the development of pathological fibrosis. Mechanically, lncRNAs can function as competing endogenous RNAs (ceRNAs), acting as miRNA sponges to disrupt the interaction of miRNAs and their target mRNAs [14]. For instance, *LINC00084* has been demonstrated to mediate myofibroblast activation in fibrotic buccal mucosa fibroblasts. The upregulation of this lncRNA increases the epithelial-to-mesenchymal transition (EMT)-activator *ZEB1* by sponging *miR-204* [15]. Another study showed that the arecoline-induced *lncRNA H19* binds *miR-29b*, thereby impeding *miR-29b* from interacting with type I collagen (*COL1A1*) and inhibiting several myofibroblast phenotypes [16]. However, the specific functions of dysregulated lncRNAs in the progression of OSF remain inadequately explored.

Myocardial infarction-associated transcript (*MIAT*), an intergenic lncRNA initially identified as a risk locus for myocardial infarction [17], has since been found to be overexpressed in various cancers, where it regulates multiple biological processes such as cell cycle, invasion, metastasis, and drug resistance [18]. In oral cancer tissues, *MIAT* is upregulated and associated with poor prognosis [19]. *MIAT* has also been implicated in several fibrotic diseases, including heart failure [20], renal fibrosis [21], and chronic pancreatitis [22]. Notably, *MIAT* knockdown has been shown to inhibit TGF-β-stimulated myofibroblast formation in mouse fibroblasts [21]. However, the role of *MIAT* in OSF development, particularly in influencing myofibroblast transdifferentiation, remains unclear. Given *MIAT*’s pro-fibrotic role, elucidating its precise molecular interactions and pathways is crucial. This study, therefore, aims to investigate *MIAT*’s involvement in myofibroblast activation and OSF progression, as well as its ceRNA network, addressing a critical gap in our understanding of OSF pathogenesis.

## RESULTS

### *MIAT* expression increases in the fibrotic buccal tissues and its derived primary fibroblasts

**Figure 1:**
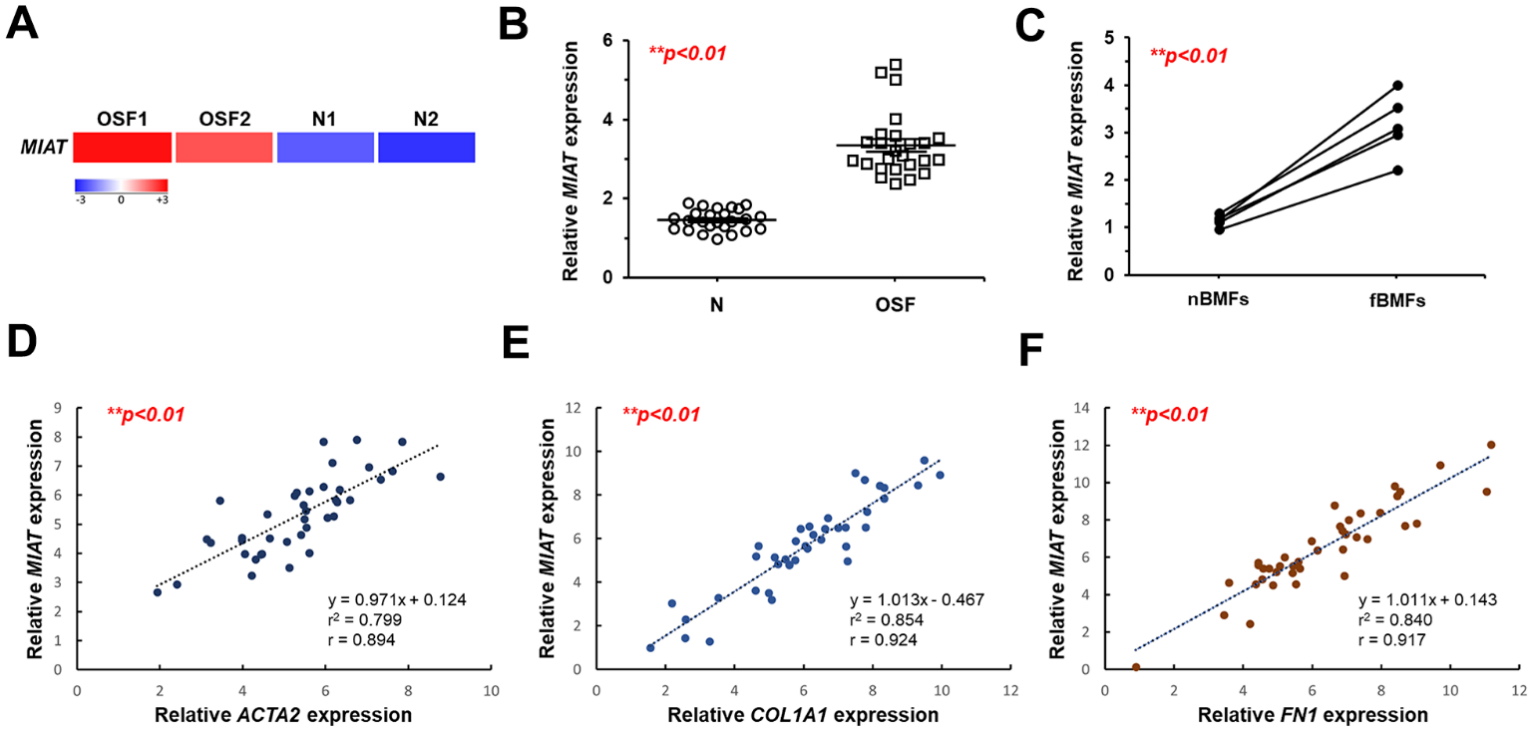
***MIAT* is upregulated in fibrotic buccal tissues and primary fibrotic buccal mucosa fibroblasts from patients with OSF.** (**A**) RNA-sequencing analysis showed that *MIAT* was an up-regulated differentially expressed gene (fold change ≥ 2.0; *p* < 0.01) in fibrotic tissue samples (OSF) compared to normal tissues (N) from patients with OSF (n=2) and healthy individuals (n=2). (**B**) The relative expression of *MIAT* in samples of normal (N; n=25) and fibrotic (OSF; n=25) tissue was assessed by qRT-PCR analysis. Data are mean ± S.D. (**C**) The relative expression of *MIAT* was further assessed in primary normal buccal mucosa fibroblasts (nBMFs; n=5) and fibrotic buccal mucosa fibroblasts (fBMFs; n=5) by qRT-PCR, with differences between groups analyzed using the paired Student’s *t*-test. (**D**–**F**) A significant positive correlation was observed between the expression of *MIAT* and fibrotic markers, including *ACTA2* (encoding α-SMA; **D**), *COL1A1* (**E**), and *FN1* (**F**) in samples of fibrotic tissue (n=40).

To identify key dysregulated lncRNAs involved in OSF progression, we initially established a cohort comprising 25 patients with OSF and 25 healthy individuals. RNA-sequencing analysis showed that *MIAT* was aberrantly upregulated in the fibrotic buccal tissues from OSF patients (OSF; n=2) compared to that of normal tissues from healthy individuals (N; n=2; Figure 1A). Results from qRT-PCR confirmed overexpression of *MIAT* in OSF specimens (n=25) and fBMFs (n=5) compared to normal tissues (N; n=25) and fibroblasts derived from non-fibrotic buccal mucosa (nBMFs; n=5), respectively (Figure 1B, 1C). These findings suggest that dysregulated MIAT expression in fibrotic tissues may be associated with myofibroblasts activation. To further validate the association between *MIAT* expression and myofibroblasts activation, we expanded our cohort to include 45 OSF patients and assessed the expression of pro-fibrotic markers in their fibrotic buccal tissues, including alpha-smooth muscle actin (α-SMA; encoded by *ACTA2*), collagen type 1 alpha 1 (*COL1A1*), and fibronectin (*FN1*). As expected, results from qRT-PCR revealed that the expression of *MIAT* was positively correlated with *ACAT2* (Figure 1D), *COL1A1* (Figure 1E), and *FN1* (Figure 1F). It has been known that myofibroblast acquires more contractile phenotype by increased actin stress fibers, so α-SMA can be used as a differentiated myofibroblast marker [23]. During differentiation, myofibroblasts display increased expression and secretion of FN and collagen [24]. As such, we postulated that the expression of *MIAT* may be involved in the regulation of myofibroblast activation and contribute to the development of OSF.

### Silencing *MIAT* reduces the myofibroblastic properties of fBMFs

**Figure 2:**
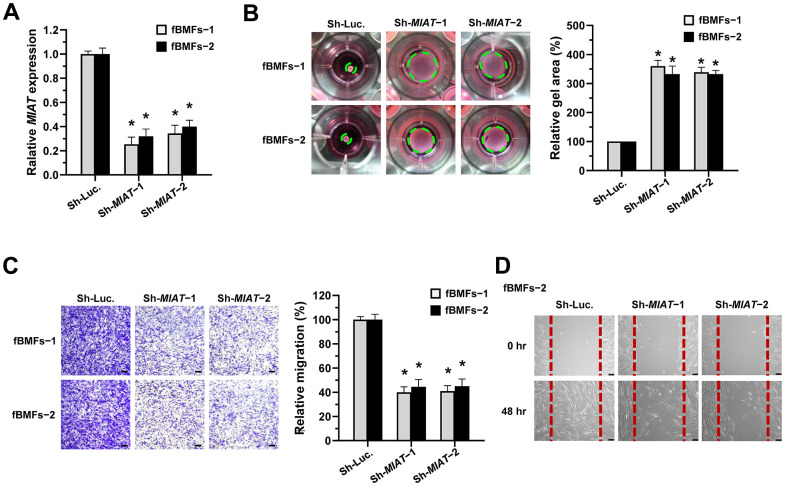
**Knockdown of *MIAT* suppresses the myofibroblastic properties.** (**A**–**D**) Primary fBMFs (obtained from two patients with OSF; fBMFs−1 and −2) were transfected with lentiviruses expressing non-targeting ShRNA (Sh-Luc.) and Sh-*MIAT* (Sh-*MIAT*−1 and −2). The *MIAT* knockdown efficiency was assessed using qRT-PCR analysis (**A**). The cells (fBMFs−1 and −2) were then cultured in collagen gel for additional 48 hours, and the gel area after cell contraction was measured (**B**). Cells (fBMFs−1 and −2) were cultured in Transwell system for an additional 24 hours, and their migration ability was quantified (**C**). Data are presented as mean ± SD (n=3); *p < 0.05 vs. Sh-Luc. (**A**–**C**). Confluent monolayers of fBMFs−2 were scratched and cultured for 48 hours, and the wound closure was assessed. Scale bar, 50 μm (**D**).

To test our presumption, we used a lentiviral vector-mediated short hairpin (sh) RNA targeting *MIAT* to knock down the expression of *MIAT* in fBMFs (Figure 2A) and examined the effect of *MIAT* on myofibroblast phenotypes. First, a collagen gel contraction assay was conducted to assess the contractile forces generated by myofibroblasts, which propagated throughout the collagen matrix and resulted in decreased matrix size. As shown in Figure 2B, the downregulation of *MIAT* markedly attenuated the collagen gel contractility of fBMFs. Another feature of myofibroblasts is that they proliferate and migrate to the damaged site during wound healing. By using Transwell migration and scratch (wound healing) assays, we demonstrated that suppression of *MIAT* significantly inhibited the cell migration (Figure 2C) and wound-healing ability (Figure 2D) of fBMFs.

### Loss of *MIAT* inhibits the arecoline-induced myofibroblast transformation of nBMFs

**Figure 3:**
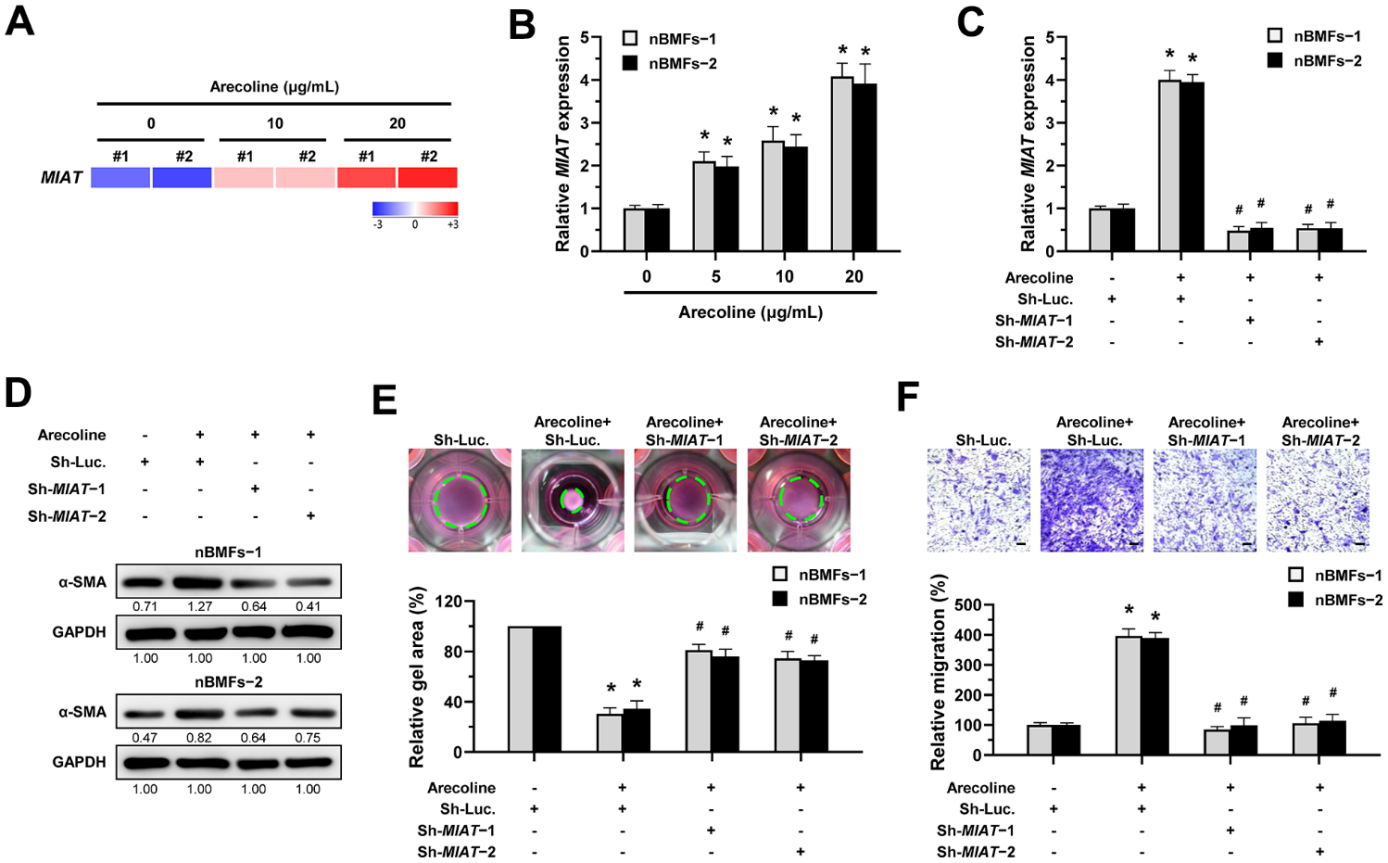
**Knockdown of *MIAT* impairs the arecoline-induced myofibroblastic transformation in BMFs.** (**A**) Primary nBMFs (obtained from two healthy individuals; nBMFs−1 and −2) were cultured with arecoline (0, 10, and 20 μg/mL) for 24 hours, followed by RNA-sequencing analysis to determine the levels of *MIAT* (*p* < 0.01). (**B**) Normal BMFs (−1 and −2) were cultured with arecoline (0, 5, 10, and 20 μg/mL) for 24 hours, followed by qRT-PCR analysis to determine the *MIAT* expression. (**C**–**F**) Normal BMFs (−1 and −2) were transfected with lentiviruses expressing non-targeting ShRNA (Sh-Luc.) and Sh-*MIAT* (Sh-*MIAT*−1 and −2). After 48 hours, the cells were cultured with or without arecoline (20 μg/mL) for 24 hours for the induction of myofibroblasts transdifferentiation. The expression of *MIAT* in each group was assessed using qRT-PCR analysis. Data are presented as mean ± SD (n=3); *p < 0.05 vs. Sh-Luc.; #*p* < 0.05 vs. Sh-Luc. with arecoline treatment (**C**). The protein expression of α-SMA in each group was determined using Western blotting analysis (**D**). Cells (nBMFs−1 and −2) were cultured in collagen gel for an additional 48 hours, and the resulting gel area after cell contraction was measured (**E**). Cells (nBMFs−1 and −2) were cultured in Transwell system for an additional 24 hours, and their migration ability was quantified (**F**). Data are presented as mean ± SD (n=3); *p < 0.05 vs. Sh-Luc.; #*p* < 0.05 vs. Sh-Luc. with arecoline treatment; Scale bar, 50 μm (**E**, **F**).

We previously demonstrated that arecoline treatment can effectively induce nBMFs to acquire myofibroblastic properties [15, 25–27]. Thus, to understand whether *MIAT* is involved in the transformation of fibroblasts to myofibroblasts, we assessed the expression of *MIAT* in nBMFs after treatment with 0, 5, 10, and 20 μg/mL arecoline for 24 hours. RNA sequencing analysis showed that *MIAT* levels were up-regulated in nBMFs treated with 10 and 20 μg/mL arecoline (Figure 3A). Also, results of qPCR analysis demonstrated that arecoline gradually increased *MIAT* expression in a dose-dependent manner (Figure 3B). Based on our previous findings [15, 25–27], in this study, nBMFs were cultured in medium containing 20 μg/mL arecoline for 24 hours. This treatment induced an increase in α-SMA expression (Figure 3D and Supplementary Figure 1), contractility (Figure 3E), and cell migration (Figure 3F), collectively confirming the acquisition of myofibroblast-like properties. As expected, the silencing of *MIAT* in the arecoline-treated nBMFs (Figure 3C) successfully attenuated the expression of α-SMA (Figure 3D), and significantly reduced both collagen gel contractility (Figure 3E) and Transwell migration capacity (Figure 3F). These results implied that downregulation of *MIAT* may prevent myofibroblast transformation and potentially mitigate the progression of OSF.

### *MIAT* promotes myofibroblastic properties by acting as a sponge of *miR-342-3p*

**Figure 4:**
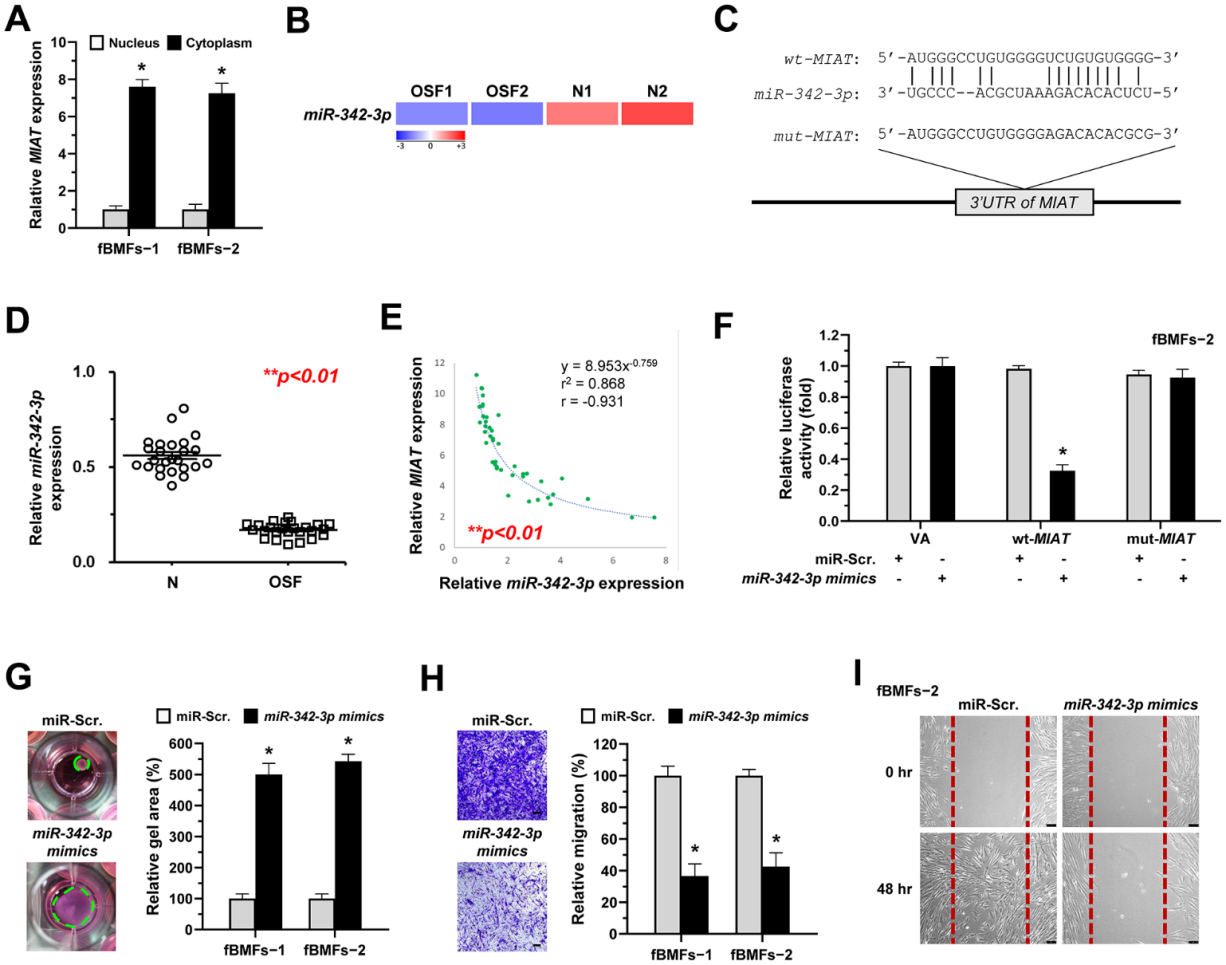
***MiR-342-3p* negatively correlates to *MIAT* expression in OSF tissues and acts as an anti-fibrotic miRNA in OSF.** (**A**) RNA of cytoplasmic and nuclear fractions from primary fBMFs (−1 and −2) were analyzed by qRT-PCR to determine the subcellular localization of *MIAT*. Data are presented as mean ± SD (n=3); *p < 0.05 vs. Nucleus. (**B**) RNA-sequencing analysis showed that *miR-342-3p* was a down-regulated differentially expressed gene (fold change ≤ -2.0; p < 0.01) in samples of fibrotic tissues (OSF; n=2) compared to normal tissues (N; n=2). (**C**) An illustration of the predicted pairing region between *miR-342-3p* and *MIAT* 3’UTR, discovered using the miRanda database, and the 3’ UTR regions of full-length (wt-*MIAT*) and mutated *MIAT* (mut-*MIAT*) complementarity to the seed site of *miR-342-3p,* predicted by TargetScan *in silico* browser. (**D**) The relative expression of *miR-342-3p* in normal (N; n=25) and fibrotic (OSF; n=25) tissues was assessed by qRT-PCR analysis. Data are presented as mean ± SD. (**E**) A significant negative correlation was observed between *MIAT* and *miR-342-3p* expression fibrotic tissue samples (OSF; n=45). (**F**) Fibrotic BMFs (−1) were co-transfected with either miR-Scramble (miR-Src.) or *miR-342-3p mimics*, along with the indicated pmirGLO-based constructs shown in (**C**). Luciferase reporter activity was measured 24 hours post-transfection. Data are presented as mean ± SD (n = 3); *p < 0.05 vs. wt*-MIAT* with miR-Src. (**G**–**I**) Fibrotic BMFs (−1 and −2) expressing Sh-Luc or Sh-*MIAT* were transfected with either miR-Src or *miR-342-3p inhibitor* for 24 hours. The cells (fBMFs−1 and −2) were cultured in collagen gel for an additional 48 hours, and the resulting gel area after cell contraction was measured (**G**). Cells (fBMFs−1 and −2) were cultured in Transwell system for an additional 24 hours, and their migration ability was quantified (**H**). Data are presented as mean ± SD (n=3); *p < 0.05 vs. miR-Scr. (**G**, **H**). Confluent monolayers of fBMF−2 were scratched and cultured for an additional 48 hours, and the wound closure was assessed (**I**). Scale bar, 50 μm.

Recent studies focused on the biological roles of lncRNAs as miRNA sponges. Due to the miRNA-mediated post-transcriptional suppression of targeted mRNA occurs in the cytoplasm, determining the subcellular localization of *MIAT* is precedence. Herein, we showed that *MIAT* was preferentially located in the cytoplasm of fBMFs (Figure 4A). By using bioinformatics prediction tools, several putative miRNA binding sites contained in *MIAT* sequences were identified, including *miR-342-3p*. Since a recent study has demonstrated the interplay between *MIAT* and *miR-342-3p* in retinal pericytes [28] and *miR-342-3p* was downregulated in OSF tissues using RNA sequencing analysis (Figure 4B), we then investigated whether *miR-342-3p* mediated the fibrosis effect of *MIAT*. Bioinformatics analysis predicted that *MIAT 3*’*UTR* sequence had a potential binding region for *miR-342-3p* (Figure 4C). To confirm the significance of *miR-342-3p* in OSF, the qRT-PCR analysis was conducted to evaluate its expression in clinical specimens. As expected, *miR-342-3p* was significantly decreased in OSF tissues (Figure 4D). Notably, we also found a significantly negative correlation between *MIAT* expression and *miR-342-3p* levels in OSF tissues (r = − 0.931, p<0.05, Figure 4E). Results demonstrated that the *miR-342-3p mimics* evidently lowered the activity in *wt-MIAT* group but had no effect in *mut-MIAT* group (Figure 4F). Furthermore, we showed fBMFs with *miR-342-3p mimics* exhibited lower myofibroblast features, including collagen gel contraction, Transwell migration and wound healing abilities (Figure 4G–4I).

**Figure 5:**
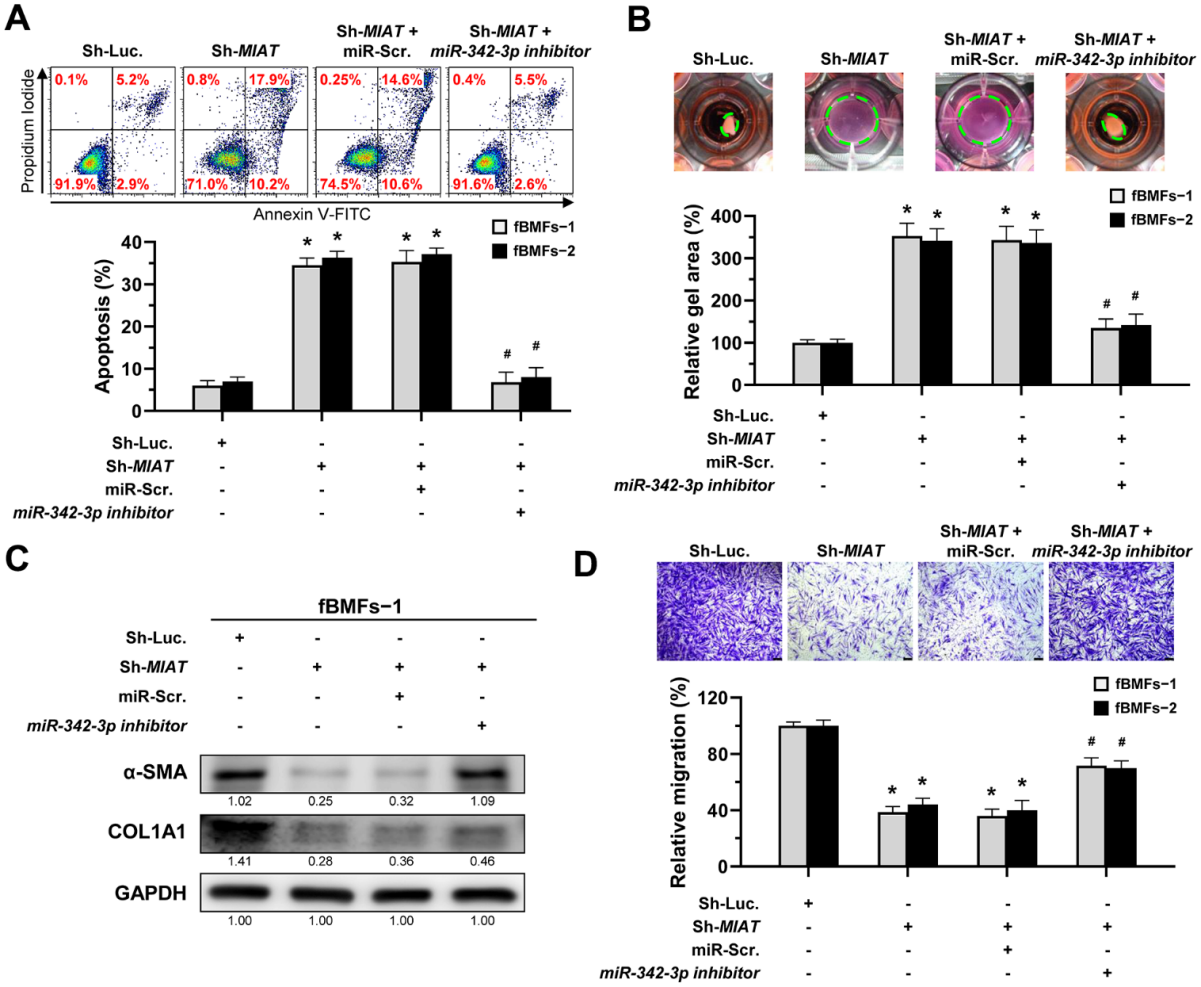
**The silencing of *MIAT* induces apoptosis and inhibits myofibroblastic properties by targeting *miR-342-3p*.** (**A**–**D**) Fibrotic BMFs (fBMFs−1 and −2) expressing the Sh-Luc. or Sh-*MIAT* were transfected with either miR-Scramble (miR-Src.) or *miR-342-3p inhibitor* for 24 hours. Cell apoptosis (annexin V^+^ or annexin V^+^/PI^+^) was assessed using flow cytometry (**A**). Cells (fBMFs−1 and −2) were cultured in collagen gel for an additional 48 hours, followed by the measurement of the gel area after cell contraction (**B**). Data are presented as mean ± SD (n=3); *p < 0.05 vs. Sh-Luc.; #*p* < 0.05 vs. Sh-*MIAT* with miR-Src. (**A**, **B**). The protein expression of α-SMA and COL1A1 in fBMFs−1 was analyzed using Western blotting (**C**). Cells (fBMFs−1 and −2) were cultured in Transwell system for an additional 24 hours, and their migration ability was quantified. Data are presented as mean ± SD (n=3); *p < 0.05 vs. Sh-Luc.; #*p* < 0.05 vs. Sh-*MIAT* with miR-Src.; Scale bar, 50 μm (**D**).

Given that *miR-342-3p* is negatively correlated with *MIAT* and that adding this miRNA to fBMFs inhibited myofibroblast differentiation, it is crucial to investigate whether *MIAT* directly sponges *miR-342-3p* in its exertion of fibrotic trait. Since the evasion of apoptosis by myofibroblasts is a hallmark of fibrotic disorders, understanding this interaction is essential [10]. Thus, we measured the percentage of apoptotic cells in fBMFs infected with *MIAT* shRNA along with *miR-342-3p inhibitor*. Our results showed that transfection of *miR-342-3p inhibitor* into fBMFs successfully avoided cell apoptosis induced by silencing of *MIAT* (Figure 5A). Similarly, *miR-342-3p inhibitor* counteracted the effect of silencing of *MIAT* on collagen gel contraction (Figure 5B), protein expression of α-SMA and COL1A1 (Figure 5C and Supplementary Figure 2), and cell migration (Figure 5D). Taken together, these data indicate that *MIAT* functions as a *miR-342-3p* sponge to promote myofibroblast properties in fBMFs.

### *MiR-342-3p* mitigates myofibroblast activation by suppressing *SOX6*

**Figure 6:**
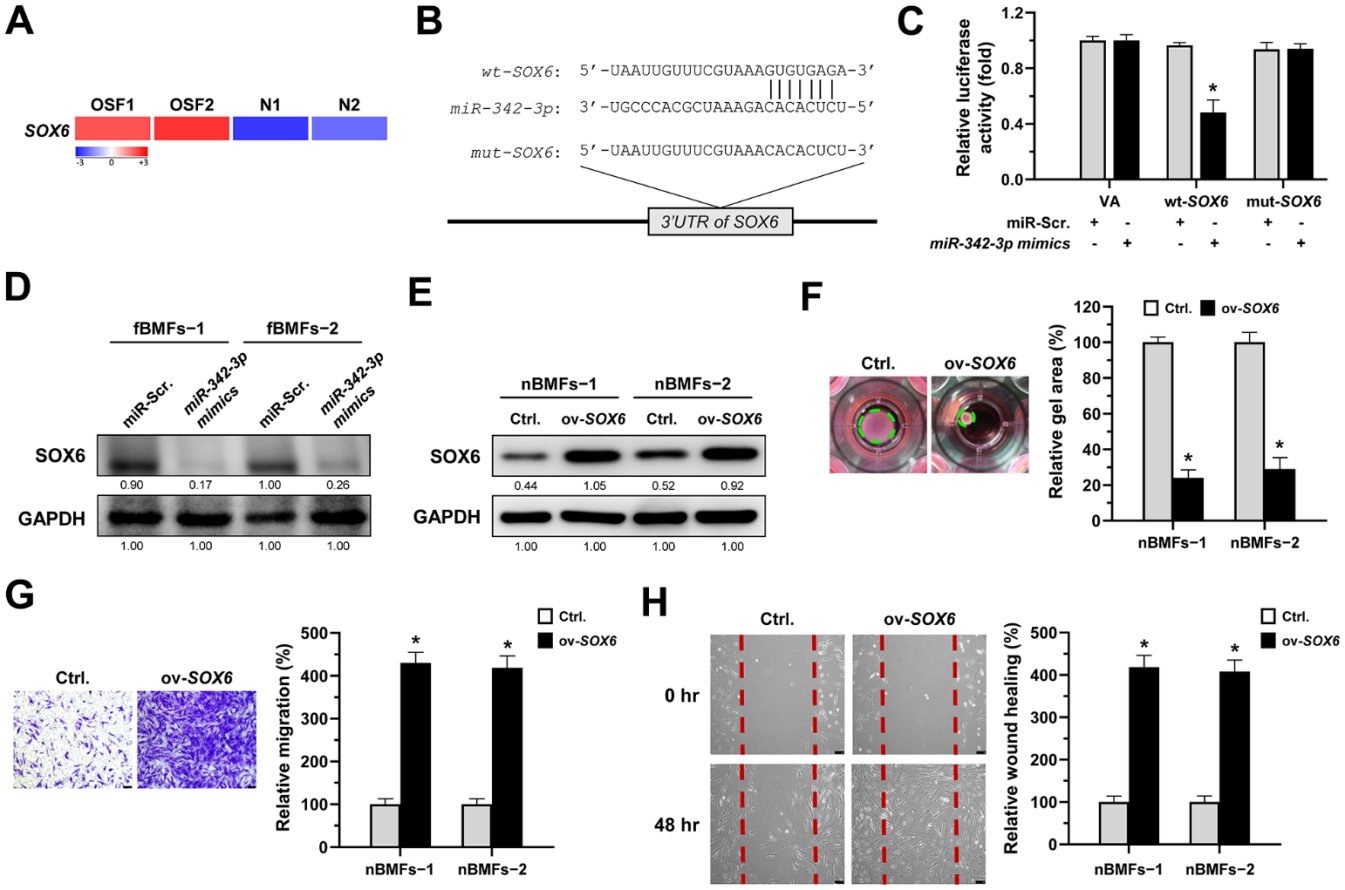
***SOX6* is a target of *miR-342-3p*.** (**A**) RNA-sequencing analysis showed that *SOX6* was an up-regulated differentially expressed gene (fold change ≥ 2.0; p < 0.01) in fibrotic tissues samples (OSF; n=2) compared to normal tissues samples (N; n=2). (**B**) An illustration of the predicted pairing region between *miR-342-3p* and *SOX6* 3’UTR were discovered using the miRDB database, and the 3’ UTR regions of full-length (wt*-SOX6*) and mutated *SOX6* (mut*-SOX6*) complementarity to the seed site of *miR-342-3p,* predicted by the TargetScan *in silico* browser. (**C**) Fibrotic BMFs (−1) were co-transfected with either miR-Scramble (miR-Src.) or *miR-342-3p mimics*, along with the indicated pmirGLO-based constructs shown in (**B**). Luciferase reporter activity was measured 24 hours post-transfection. Data are presented as mean ± SD (n = 3); *p < 0.05 vs. wt*-SOX6* with miR-Src. (**D**) Fibrotic BMFs (−1 and −2) were transfected with either miR-Src or *miR-342-3p inhibitor* for 24 hours, followed by Western blotting analysis to determine the protein expression of SOX6. (**E**–**H**) Normal BMFs (−1 and −2) were transfected with lentiviruses expressing control vector (Ctrl.) or *SOX6* (ov-*SOX6*). The overexpression efficiency of *SOX6* was assessed by Western blotting analysis (**E**). Cells (nBMFs−1 and −2) were cultured in collagen gel for an additional 48 hours, and the resulting gel area after cell contraction was measured (**F**). Cells (nBMFs−1 and −2) were cultured in Transwell system for an additional 24 hours, and their migration ability was quantified (**G**). Confluent monolayers of cells (nBMFs−1 and −2) were scratched and cultured for an additional 48 hours, and the wound closure was assessed (**H**). Data are presented as mean ± SD (n=3); *p < 0.05 vs. Ctrl. (**F**–**H**). Scale bar, 50 μm.

Several studies have shown that *SRY-box transcription factor 6 (SOX6)* may be a target of *miR-342-3p* in renal or cardiac injuries [29, 30] and implicate in renal fibrosis [29, 31], so we sought to examine whether *SOX6* plays a role for the anti-fibrosis effect of *miR-342-3p* during oral fibrogenesis. By using RNA sequencing analysis, we showed that *SOX6* was upregulated in OSF tissues compared to normal specimens (Figure 6A). We also predicted a potential *miR-342-3p* binding region on the *SOX6* 3’UTR sequence via miRDB bioinformatic analysis (Figure 6B), and the results from a luciferase assay showed that the luciferase activity was significantly reduced in *wild-type SOX6* in nBMFs (*wt-SOX6*), while there was no change in *mutated SOX6* (*mut-SOX6*, Figure 6C). Besides, the expression of SOX6 in fBMFs was downregulated after transfection of *miR-342-3p mimics* (Figure 6D and Supplementary Figure 3). Additionally, we showed that overexpression of SOX6 in nBMFs (Figure 6E and Supplementary Figure 4) elicited multiple myofibroblast phenotypes, including higher collagen gel contractility (Figure 6F), Transwell migration (Figure 6G) and wound healing (Figure 6H) capacities. These results supported that *miR-342-3p* suppresses myofibroblastic properties by targeting *SOX6*.

### *MIAT* promotes myofibroblastic properties through the *miR-342-3p/SOX6* axis

**Figure 7:**
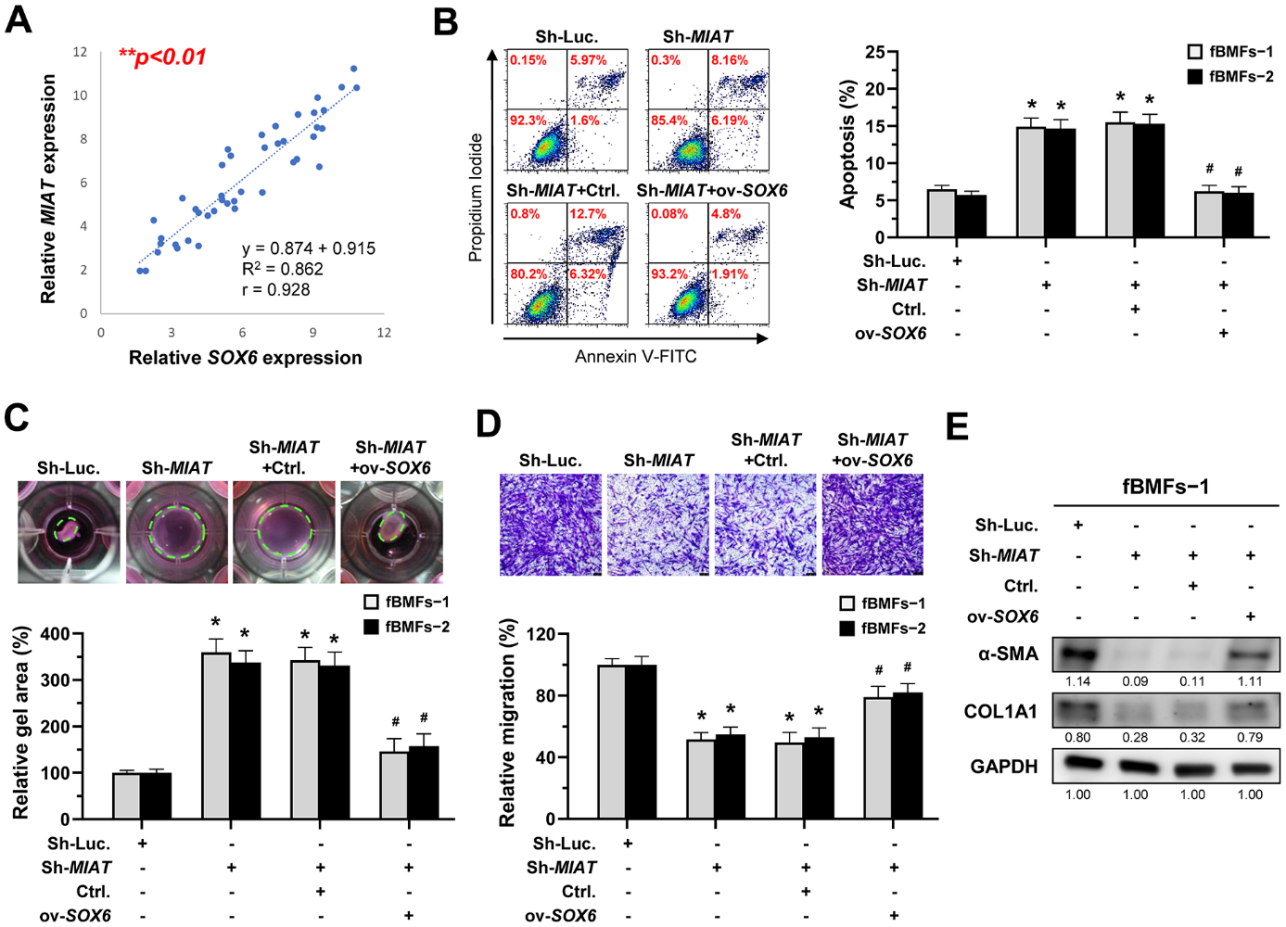
***MIAT* increases the myofibroblastic properties by positively regulating *SOX6*.** (**A**) A significant positive correlation between the expression of *MIAT* and *SOX6* in fibrotic tissue sample (OSF; n=43). (**B**–**E**) Fibrotic BMFs (−1 and −2) were co-transfected with lentiviruses expressing the following constructs in the indicated combinations: non-targeting ShRNA (Sh-Luc.), Sh-*MIAT*, control vector (Ctrl.), and *SOX6* (ov-*SOX6*). Cell apoptosis (annexin V^+^ or annexin V^+^/PI^+^) was determined using flow cytometry (**B**). Cells (fBMFs−1 and −2) were cultured in collagen gel for an additional 48 hours. The resulting gel area after cell contraction was measured (**C**). Cells were cultured in Transwell system for an additional 24 hours, and their migration ability was assessed. Scale bar, 50 μm (**D**). Data are presented as mean ± SD (n=3); *p < 0.05 vs. Sh-Luc.; #*p* < 0.05 vs. Sh-*MIAT* with Ctrl. (**B**–**D**). The protein expression of α-SMA and COL1A1 in fBMFs−1 was analyzed using Western blotting (**E**).

**Figure 8:**
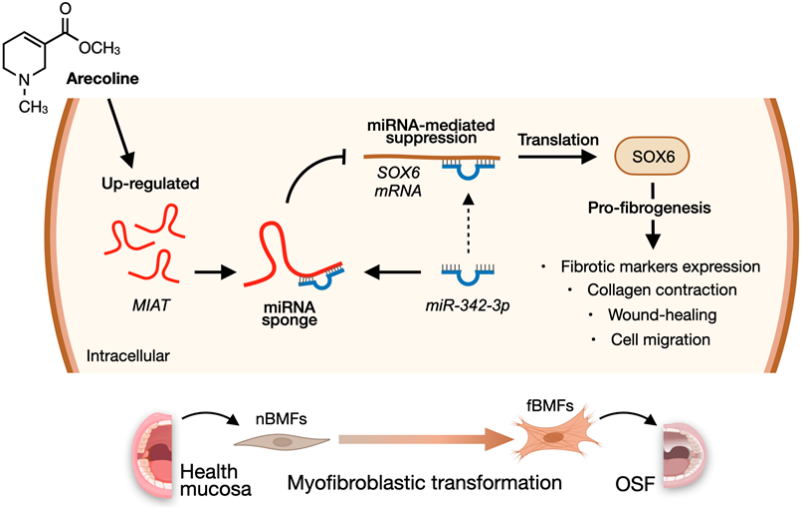
**A diagram illustrates the mechanism of *MIAT* in the development of OSF.** Upregulation of MIAT in nBMFs following chronic exposure to arecoline may interfere the miR-342-3p-mediated suppression of SOX6, resulting in persistent activation of fBMFs and development of OSF.

According to the above findings that *miR-342-3p* can bind to *MIAT* and *SOX6 mRNA*, we speculated that *MIAT* may serve as a ceRNA to regulate the *miR-342-3p*/*SOX6* axis during OSF progression. To verify this hypothesis, we assessed the relationship between *MIAT* and *SOX6* and found they were positively correlated (Figure 7A). Furthermore, our results demonstrated that overexpression of *SOX6* blocked the effect of *MIAT* silencing on apoptosis (Figure 7B), collagen gel contraction (Figure 7C), Transwell migration (Figure 7D) and the expression of fibrosis markers (Figure 7E and Supplementary Figure 5). Altogether, our data indicate that upregulation of *MIAT* in nBMFs following chronic exposure to arecoline may interfere the *miR-342-3p*-mediated suppression of *SOX6*, resulting in persistent activation of fBMFs and development of OSF (Figure 8).

## DISCUSSION

*MIAT*, also termed as *Gomafu* or *LINC00066*, is located at 22q12.1 with a length of 30,051 bp and was first identified in 2000 [32]. Ishii et al*.* later found *MIAT* is located within a susceptible locus for myocardial infarction (MI), leading them to name this novel gene as *MIAT* [17]. Beyond its role in cardiovascular diseases [33–35], MIAT dysregulation has been associated with a variety of disorders, such as impaired neuronal function [36] and glioblastoma [37] and various fibrosis diseases. For instance, *MIAT* has been shown to modulate cardiac fibrosis by serving as a ceRNA and negatively regulating multiple miRNAs, such as miR-29a-3p [38], miR-24 [39], or miR-214-3p [40]. Moreover, TGF-β1-induced *MIAT* contributes to the activation of pancreatic stellate cells in chronic pancreatitis and proximal tubule epithelial cells in renal interstitial fibrosis by targeting miR-216a-3p/COX-2 [22] or miR-145/EIF5A2 axes [41], respectively. In this study, we showed that *MIAT* was upregulated in OSF specimens, and the silencing of MIAT reduced myofibroblast phenotypes and activation. Our results further demonstrated that the arecoline-stimulated *MIAT* conferred myofibroblast transdifferentiation of nBMFs by acting as a ceRNA for *miR-342-3p*, thereby alleviating its repression on *SOX6*.

Aberrant expression of *miR-342* has been found in various diseases, such as diabetic nephropathy [29, 42] or cardiomyopathy [43]. Unlike most miRNAs where only one guide strand is loaded into RNA-induced silencing complex (RISC) while the other strand is destroyed rapidly, both *miR-342-3p* and *miR-342-5p* are excised from the same stem-loop precursor miRNA and become mature miRNAs [44]. It has been revealed that *miR-342-3p* acts as a tumor suppressor in oral cancer by inhibiting LIM and SH3 protein 1 [45], and our results suggested that dysregulation of *miR-342-3p* also participated in the development of precancerous OSF. Several studies have demonstrated the involvement of *miR-342* in numerous fibrosis diseases by influencing myofibroblast activation. For example, *miR-342-3p* was found to regulate hepatic stellate cell (HSC) activation by affecting the Zbtb7a-mediated TGF-β signaling in a model of *Echinococcus multilocularis* infected liver fibrosis [46]. Another study showed that *miR-342* directly interacted with Sp1 and inhibited its downstream TGF-β1/Smad signaling, leading to the inhibition of HSCs activation [47]. In renal fibrosis, *miR-342-5p* has been revealed to target Ptch1 and inhibit its transcription factor FoxO3, leading to autophagy in the TGF-β1-stimulated TCMK-1 (mouse kidney) cells [48]. *MiR-342-3p* also has been demonstrated to increase cell proliferation and inhibit apoptosis of renal mesangial cells by reducing *SOX6* expression [29]. In line with this finding, we showed that *miR-342-3p* exhibited anti-fibrosis properties in fBMFs, possibly through direct suppression of *SOX6*.

*SOX6* is a transcription factor that was discovered in 1990s [49, 50] and belongs to the *SOXD* subfamily along with *SOX5* and *SOX13* [51]. In humans, *SOX6* and *SOX5* are located in paralogous chromosomal regions on 11p15.3–15.2 and 12p12.1, respectively. Although *SOX6* do not harbor any transactivation or transrepression domains, it has been found to bind to various proteins, cofactors, and miRNAs to regulate the transcription and functions of multiple genes [52–54]. Several studies suggested that differential expression of *SOX6* may account for numerous diseases, such as cardiomyopathy [54, 55]. In fact, emerging evidence demonstrated that *SOX6* serves as a target for a number of miRNAs in renal fibrosis such as *miR-342-3p* [29], *miR-19b* [56], and *miR-185-5p* [31]. Notably, these studies showed that miRNA-mediated sponging of *SOX6* promotes cell apoptosis and reduces the expression of fibrosis markers like fibronectin and type I collagen [29, 31]. Our results are consistent with these findings, showing that SOX6 suppresses apoptosis in myofibroblasts, thereby increasing fibrosis markers. Furthermore, we demonstrated that manipulation of *SOX6* affected myofibroblast marker (α-SMA) expression and phenotypes, which provided direct evidence that upregulation of *SOX6* leads to oral fibrogenesis through modulation of myofibroblast transdifferentiation.

A limitation of this study is the inconsistency in sample sizes between the experiments analyzing gene expression correlations (e.g. Figure 1D–1F; n=40) and those comparing gene expression differences between OSF and normal (N) groups (e.g. Figure 1B; n=25). To minimize potential confounding variables, we utilized samples from patients with OSF and healthy individuals recruited during the same period (n=25 per group) when comparing the expression differences of target genes. To enhance the statistical significance of our results, we continued to recruit OSF patients, expanding the sample size from 25 to 45. Our association analysis has indicated that the expression of *MIAT* strongly correlated to each gene of interest (Figures 1D–1F, 4E, 7A), suggesting that similar findings could be obtained from the smaller sample size (n=25). However, it is necessary to continue expanding both the number and diversity of patients, such as genetic background and clinical stage of disease progression. This would be beneficial to strengthen the evidence base of our study and to further elucidate the role of *MIAT* in OSF pathogenesis.

In conclusion, our findings revealed that the aberrantly overexpressed *MIAT* in OSF tissues may be due to chronic stimulation of arecoline, resulting in transdifferentiation of nBMFs via titrating the inhibitory effect of *miR-342-3p* on *SOX6* expression. These results not only offered insight into how upregulated *MIAT* led to OSF, but also demonstrated that targeting this *MIAT/miR-342-3p*/*SOX6* pathway may be a promising treatment direction.

## MATERIALS AND METHODS

### Tissue preparation

45 samples of fibrotic buccal mucosa tissues (OSF) were obtained from surgical resection of OSF patients who did not receive any preoperative treatment at the Department of Dentistry, Chung Shan Medical University Hospital; and 25 samples of normal buccal mucosa tissues (N) were obtained from the surgical removal of impacted third molars of healthy individuals. All samples were obtained with the written informed consent of patients. For RNA-sequencing and qRT-PCR analysis, the obtained samples were immediately stored at -80° C before use. All ethical regulations and operations were complied with Institutional Review Board of Chung Shan Medical University Hospital. The histopathological identification of OSF samples was verified by two pathologists independently. Tissue specimens from 25 OSF patients and 25 healthy individuals, initially recruited during the same period, were used to analyze the statistical differences in the expression levels of various genes between the OSF and normal groups. Specimens from all recruited OSF patients (n = 45, including the initial 25 and an additional 20 subsequently recruited) were used to analyze the correlations among the expression levels of various genes of interest. Due to the limited availability of clinical tissue samples, the actual number of samples used in the experiments ranged from 40 to 45 (specific sample sizes are indicated in the respective figure legends).

### Cell culture

Fibrotic buccal mucosa fibroblasts (fBMFs) and normal BMFs (nBMFs) were extracted from buccal mucosa tissues of OSF patients and healthy individuals, respectively. The details of primary cell isolation and culture were previously described [16]. In brief, buccal mucosa tissues obtained from surgery were incubated in Hanks’ Balanced Salt Solution (HBSS) at 4° C and transferred to the laboratory immediately for further processing. After trypsinization, the tissues were cultured in DMEM medium (with 10% fetal bovine serum [FBS], and 1% penicillin-streptomycin cocktail) and plated into 25-T flasks for 14 days. The cells which are spindle-shaped and migrated out of the tissues were defined as buccal fibroblasts. All cells were continuously passaged and used for subsequent experiments between the 3rd and 8th passages and were negative for mycoplasma contamination verified using short tandem repeat (STR) DNA profiling. Arecoline was purchased from Sigma (St. Louis, MO, USA). Arecoline was dissolved in phosphate-buffered saline (PBS) as a stock solution stored at -20° C before use. When nBMFs reached a designated density, cells were treated with arecoline at a series concentration (0-20 μg/mL) for 24 hours.

### RNA-sequencing analysis

Total RNA was extracted from the collected buccal tissue samples and primary BMF cells using Trizol reagent as directed by the manufacturer (Invitrogen, Carlsbad, CA, USA). RNA quality and quantity were evaluated using NanoDrop (Thermo Fisher Scientific, Waltham, MA, USA). All procedures of RNA library preparation, sequencing, and transcriptome discrepancies analysis were performed at Genomics Inc. The detail was described in our previous studies [57]. To verify the RNA-sequencing results, the selected differentially expressed gene expressions were further confirmed by qRT-PCR analysis.

### QRT-PCR analysis

The methods of RNA extraction, quality control and quantitation are described above. For *MIAT* subcellular localization detection, the nuclear and cytoplasmic RNA from fBMFs were isolated using a PARIS™ Kit (Thermo Fisher Scientific). cDNA was prepared using The Superscript III first-strand synthesis system (Invitrogen) to reverse-transcribe RNA. ABI StepOne™ Real-Time PCR Systems (Applied Biosystems, Waltham, MA, USA) were used for PCR experiments with the resultant cDNAs. The specific primer sequences are listed as follows (5’-3’): *MIAT*, TATTTGCAGGGGGTGCTCTG (forward), GGGCAGGGGGTCTAACTCTA (reverse); *α-SMA*, AGCACATGGAAAAGATCTGGCACC (forward), TTTTCTCCCGGTTGGCCTTG (reverse); *COL1A1*, GATTCCCTGGACCTAAAGGTGC (forward), AGCCTCTCCATCTTTGCCAGCA (reverse); *FN1*, ACTGCGAGAGTAAACCTGAAGC (forward), GCGGTTTGCGATGGTACAGCT (reverse); *miR-342-3p*, GTGCTATCTGTGATTGAGGGA (forward), CGGGTGCGATTTCTGTG (reverse); *SOX6*, GTCGCTTAATGTGTGGCTCG (forward), TGTTCTTCCTGCCCTGACATT (reverse); *GAPDH*, GTGGCTGGCTCAGAAAAAGG (forward), GGGGAGATTCAGTGTGGTGG (reverse); *U6*, CTCGCTTCGGCAGCACA (forward), AACGCTTCACGAATTTGCGT (reverse). Relative expression levels were normalized using *GAPDH* as the internal control for total and cytoplasmic RNA, while *U6* served as the internal control for nuclear RNA and miRNA normalization, and the 2-ΔCt method was applied.

### Knockdown of *MIAT*

A lentiviral pLKO-shRNA-expressing vector was purchased from the National RNAi Core Facility (Academia Sinica, Taipei, Taiwan). Lentivirus particle production was performed in 293T cells by co-transfecting the pLKO-shRNA-expressing vectors with packaging and envelope plasmids with a ratio of 10:10:1 using Lipofectamine 2000 reagent (Invitrogen). The virus-containing supernatant was collected after 48 post-transfection and filtered through a 0.45-μm PVDF filter. Fibrotic BMFs were cultured with the filtered virus-containing supernatant supplemented with 8 μg/mL polybrene (Merck, Darmstadt, Germany) for 48 hours. The lentivirus-infected cells were selected by puromycin. Quantitative RT-PCR analysis was performed to verify the knockdown efficiency. The sequences for Sh-*MIAT* are listed below: *Sh-MIAT-1*, AAAAGCAGTCCAGGGTCTATTTATTGGATCCAATAAATAGACCCTGGACTGC; *Sh-MIAT-2*, AAAAGGGTTTGAACCTTTAGGATTTGGATCCAAATCCTAAAGGTTCAAACCC. A pLKO-luciferase-expressing vector was used as the non-targeting control (Sh-Luc.).

### Collagen gel contraction assay

A total of 0.5 mL of the collagen solution (2 mg collagen/mL) was mixed with 1 × 10^5^ cells and added into each well of a 24−well plate. After gel polymerization by 30 minutes of incubation at 37° C, 0.5 mL DMEM medium was added to cover the polymerized gels entirely. Then the gels were scraped out and plated into another 24−well plate for 48 hours of incubation at 37° C. Images of the contracted collagen gels were captured and quantified using ImageJ software (NIH). The area of the contracted gel was measured and compared to the initial area to calculate the percentage of contraction. A decrease in relative gel area represents an increase in the gel contraction ability of cells.

### Transwell migration assay

A total of 0.4 mL of a serum-free DMEM containing 1 × 10^5^ cells was added into the upper 8-μm pore insert, then the lower chamber was filled with 0.6 mL DMEM containing 10% FBS. After incubation at 37° C for 24 hours, non-migrated cells on the upper side of the insert membrane were removed, and the migrated cells on the lower side were fixed with cold-100% methanol for 30 minutes at room temperature. The fixed cells were stained with 0.1% crystal violet solution for 20 minutes. Migrated cells were visualized by capturing images using a microscope. For the evaluation of migrated cells, 0.4 mL of 30% acetic acid was added to each insert and incubated for 15 minutes to dissolve the crystal violet stain. The absorbance value at 570 nm of crystal violet stain from each insert was measured using a microplate reader (Infinite M200 Pro, Tecan Group Ltd., Männedorf, Germany) and quantified according to the manufacturer’s protocols.

### Wound healing assay

After cell confluence reached 90%, a straight wound was introduced across the center of each well using a 200 μL sterile pipette tip. Each well was washed with PBS to remove any detached cells and debris, and cells were cultured with a serum−free DMEM medium for 48 hours. Images of the wound area were captured at the time points of 0 and 48 hours by microscope.

### Western blot analysis

In brief, whole-cell lysate containing 20 μg of protein was separated by sodium dodecyl sulfate-polyacrylamide gel electrophoresis and then transferred onto a polyvinylidene fluoride membrane. After blocking for non-specific sites with 5% bovine serum albumin (BSA), the membrane was incubated with primary antibodies for 16 hours at 4° C, and subsequently incubated with the corresponding HRP-conjugated secondary antibody for 1 hour at room temperature. The signals of antibody binding were developed by ECL substrate (Merck) and then captured using the LAS−1000 plus analyzer (GE Healthcare, Piscataway, NJ, USA). GAPDH was used as an internal reference. All the antibodies were purchased from Cell Signaling Inc. (Danvers, MA, USA).

### *MiR-342-3p* knockdown and overexpression

*MiR-342-3p mimics*, *miR-342-3p inhibitor*, and miR-scramble negative control (miR-Scr.) were purchased from Applied Biosystems (Waltham, MA, USA), and a total of 100 nM oligonucleotide sequences were transfected into cells using Lipofectamine 2000 (Invitrogen). After 48 hours of transfection, cells were collected for subsequent experiments.

### Luciferase reporter activity assay

The binding of *miR-342-3p* to *MIAT* or *SOX6* was examined using a luciferase reporter activity assay (Promega, Madison, WI, USA). *MIAT* and *SOX6* 3’-UTR with full-length (wild-type, wt) and mutant-type (mut) were synthesized and cloned into pmirGLO vectors according to the manufacturer’s protocols. Luciferase vectors were mixed with *miR-342-3p mimics* and miR-scramble (miR-Src.), respectively, and the mixtures were then co-transfected into cells using Lipofectamine 2000 reagent. The activity of luciferase reporter was detected using the Luciferase Assay System according to the manufacturer’s instructions. The relative activity of each group was normalized with Renilla luciferase internal control (VA).

### Apoptosis assay

Briefly, 2 × 10^5^ cells were incubated with stained with 1.5 μl Annexin V-FITC reagent and 1.5 μl propidium iodide (PI) for 5 minutes at room temperature in the dark. The stained cells were then directly measured using a Calibur flow cytometer (Becton Dickinson, San Jose, CA, USA). Data were analyzed using FlowJo software v10 (FlowJo LLC, Ashland, OR, USA). The Annexin single-positive and Annexin/PI double-positive populations were considered apoptotic cells.

### Overexpression of *SOX6*

The *SOX6* cDNA was cloned into the pCDHI-MCS1-EF1-CopGFP plasmid (System Biosciences, Mountain View, CA, USA). The pCDH and two helper plasmids (packaging and envelope plasmids) were co-transfected into 293T cells using Lipofectamine 2000 reagent to produce lentiviral particles. The conditions for lentivirus production and infection were as described in the Knockdown of *MIAT* section. Following lentivirus infection, GFP-positive cells were sorted using flow cytometry. Overexpression of SOX6 was confirmed using qRT-PCR and Western blot analysis. An empty pCDHI-MCSI-EF1-COpGFP vector was used as the control group (Ctrl.).

### Statistical analysis

All results were plotted as mean ± standard deviation (S.D.). A *p*-value of 0.05 or less was considered statistically significant. Differences between two groups were analyzed using paired or unpaired Student’s *t*-test, while differences among multiple groups were analyzed using one-way analysis of variance (ANOVA). The correlation between two genes’ expression in clinical specimens was obtained using Pearson correlation analysis. All analyses were performed using Prism software, version 9.0 (Graph-Pad Inc., La Jolla, CA, USA) and SPSS software, version 21.0 (SPSS Inc., Chicago, IL, USA).

## Supplementary Material

Supplementary Figures
