# Supplementary material for: MIAT promotes myofibroblastic activities and transformation in oral submucous fibrosis through sponging the miR-342-3p/SOX6 axis
Source: Aging (Albany NY). 2024 Oct 7;16(19):12909–27. doi: 10.18632/aging.206121 (PMC11501384; doi:10.18632/aging.206121)
Supplement: Supplementary Figures [file aging-16-206121-s001.pdf]

SUPPLEMENTARY FIGURES

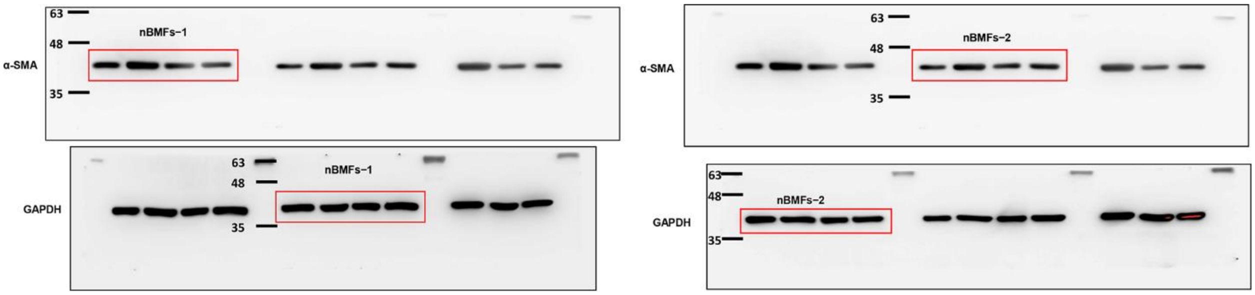

Supplementary Figure 1. Original immunoblotting data for Fig. 3D

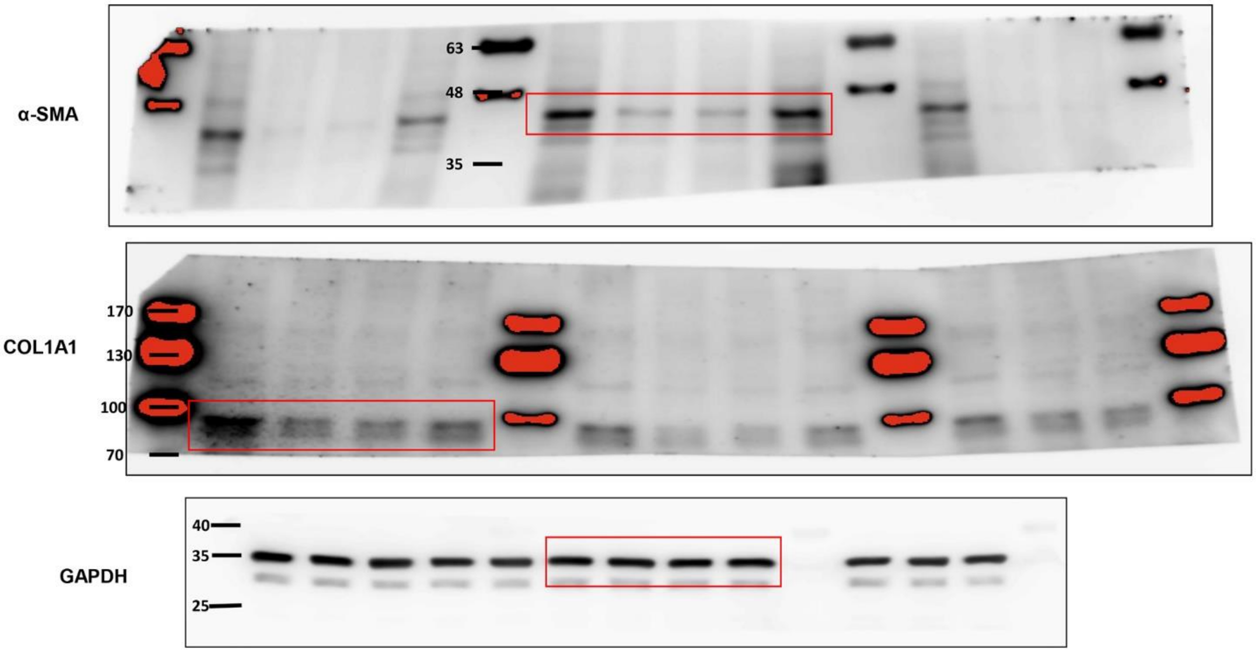

Supplementary Figure 2. Original immunoblotting data for Fig. 5C

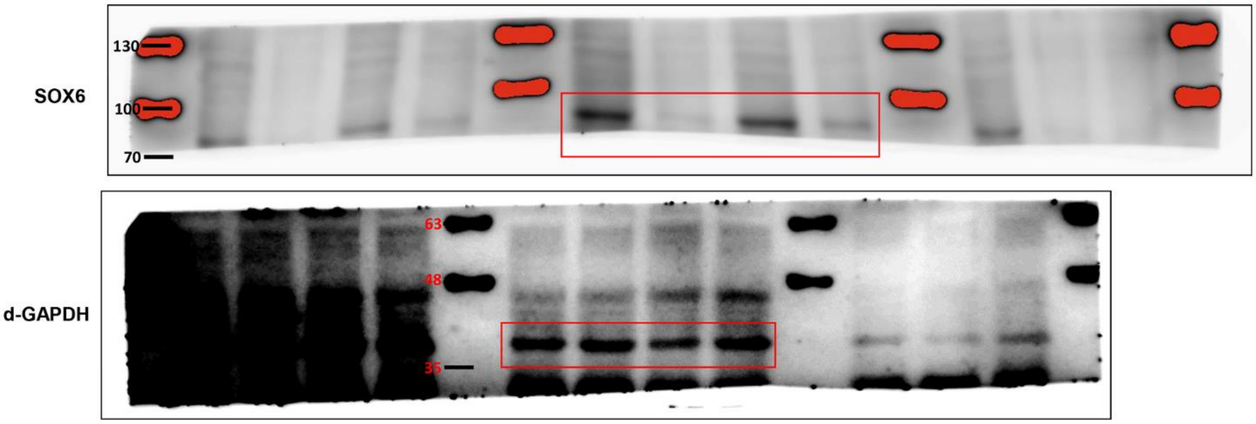

Supplementary Figure 3. Original immunoblotting data for Fig. 6D

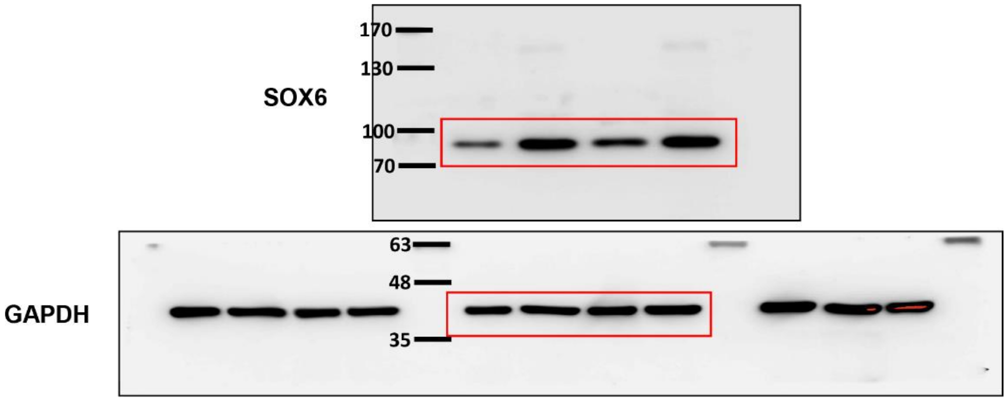

Supplementary Figure 4. Original immunoblotting data for Fig. 6E

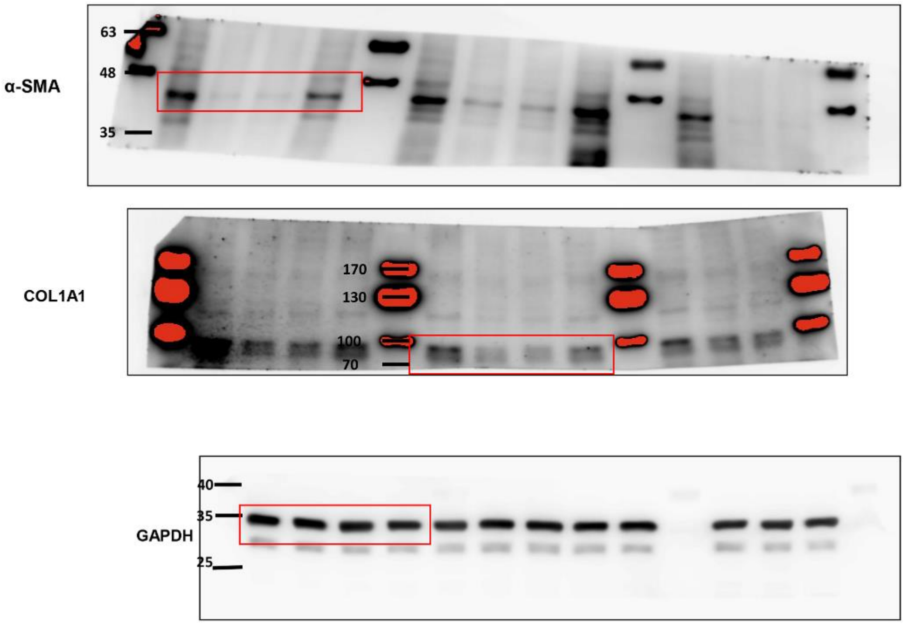

Supplementary Figure 5. Original immunoblotting data for Fig. 7E
